# Efficient and reversible Cas13d-mediated knockdown with an all-in-one lentivirus-vector

**DOI:** 10.3389/fbioe.2022.960192

**Published:** 2022-09-15

**Authors:** Suli Lv, Xuefeng Zhao, Xianyun Ma, Qingli Zou, Neng Li, Yingying Yan, Lidong Sun, Tanjing Song

**Affiliations:** ^1^ Department of Biochemistry and Molecular Biology, School of Basic Medicine, Tongji Medical College, Huazhong University of Science and Technology, Wuhan, China; ^2^ Cell Architecture Research Institute, Huazhong University of Science and Technology, Wuhan, Hubei, China

**Keywords:** Cas13d, lentivirus, CasRX, CRISPR, Tet-ON, SV40, U6

## Abstract

Type VI CRISPR effector Cas13d from Ruminococcus flavefaciens XPD3002 (RfxCas13d) is an RNA-guided RNA endonuclease. RfxCas13d has been harnessed to knockdown gene expression with high specificity in various systems including mammalian cells. While inducible knockdown is advantageous over constitutive knockdown in many scenarios, current inducible systems of RfxCas13d express CRISPR RNA and Cas13d separately. Such systems could be cumbersome to handle and may hamper the application of RfxCas13d in some scenarios. Here, we design an all-in-one Cas13d lentivirus vector which renders efficient and inducible knockdown in a doxycycline dosage-dependent manner. Furthermore, we find that Cas13d has a short half-life in mammalian cells. As a result, knockdown can be promptly reversed after doxycycline withdrawal. This vector is particularly useful for applications involving indispensable genes and/or in cells hard to transduce.

## Introduction

Being able to tune down the expression of a target gene has empowered modern genetics in multi-cellular organisms. Many methods were developed for such applications, such as anti-sense RNA, siRNA, or gene knockout. Before the era of CRISPR, siRNA was the dominating method for gene knockdown for 2 decades, and it is still widely used. Yet, the off-target effect of siRNA was not unrecognized (Seok et al., 2018), which may be attributed to the involvement of proteins critical for endogenous processes. CRISPR/Cas9-based methods, since their first application in the mammalian system (Cong et al., 2013; Mali et al., 2013), quickly gained popularity, which wipes out gene expression at the DNA level. CRISPR-based effectors are nucleases that can target and cut substrate DNA or RNA when activated by base-pairing between the target and small CRISPR-RNA. Among CRISPR effectors, Cas9 is a DNA nuclease. Unlike siRNA, CRISPR-based technologies are orthogonal to mammalian endogenous processes as they harness the immune system of microorganisms. Yet the change in DNA sequence introduced by conventional Cas9-mediated knockout is irreversible. In addition to Cas9, several other CRISPR-based single-molecule effectors have been uncovered, some of which are RNA-guided RNA nucleases, such as Cas13a (Abudayyeh et al., 2017), Cas13b (Smargon et al., 2017), and Cas13d (Konermann et al., 2018; Yan et al., 2018). These effectors are targeted to RNA by a single CRISPR RNA (crRNA) which base-pairs with the target. When used for gene knockdown, all Cas13 are reported to have fewer off-target effects than RNA interference (Abudayyeh et al., 2017; Konermann et al., 2018). Among Cas13 effectors, RfxCas13d is reported to have better efficacy than others for both protein-coding genes and non-coding genes in both cell culture and *in vivo* (Kushawah et al., 2020; Wessels et al., 2020; Li et al., 2021). As a result, Cas13d has been promptly applied to many organisms including plants (Mahas et al., 2019), mice (Zhou et al., 2020), flies (Buchman et al., 2020), and fish (Kushawah et al., 2020).

To deliver an expression cassette into mammalian cells, lentivirus vectors have many advantages including high transduction efficiency, wide-spectrum host cell types, and large packaging capacity. While the initial application of RfxCas13d utilized two vectors expressing CasRx and crRNA, respectively (Konermann et al., 2018), the two cassettes can be expressed in one lentivirus vector (Wessels et al., 2020). Yet, an inducible system would be preferred under many circumstances, for example, to study the instant effect of gene knockdown, to turn off and on genes dynamically, or to study the dosage effect of a gene. The current inducible CasRx lentivirus vector expresses crRNA and CasRx with separate vectors possibly due to the size limit of the vector (Wessels et al., 2020). Here we present an all-in-one vector design, pLenti-TRE-CasRx-U6-gRNA, which renders efficient inducible knockdown with CasRx.

## Materials and methods

Key reagents and resources used in this study are as follows.

**Table:** 

**Reagent or resource**	**Source**	**Identifier**
Antibodies		
Rabbit monoclonal anti-β-ACTIN	ABclonal	CAT# AC026; RRID:AB_2768234
Mouse monoclonal anti-HA tag	Covance	CAT# MMS-101P; RRID:AB_2314672
Rabbit monoclonal anti-HA tag	CST	CST# 3724; RRID:AB_1549585
Rabbit monoclonal anti-EZH2	CST	CST# 5246; RRID:AB_10694683
Rabbit monoclonal anti-KDM1B	CST	CST #54576; RRID:AB_2799465
Rabbit monoclonal anti-DOT1L	CST	CAT# 77087S; RRID:AB_2799889
Rabbit polyclonal anti-FKBP8	Proteintech	CAT# 11173-1-AP; RRID:AB_10597097
Rabbit monoclonal anti-OGT	CST	CAT# 24083S; RRID:AB_2716710
Mouse monoclonal H3K27me3	Abcam	CAT# ab6002; RRID:AB_305237
Rabbit monoclonal anti-METTL3	CST	CAT# 96391; RRID:AB_2800261
HRP goat aAnti-rabbit IgG (H+L) secondary antibody	Abclonal	CAT# AS014; RRID:AB_2769854
HRP goat anti-mouse (H+L) secondary antibody	Abclonal	CAT# AS003; RRID:AB_2769851
Alexa Fluor 488-AffiniPure donkey anti-rabbit IgG (H+L)	Invitrogen	CAT# 711-545-152; RRID:AB_2313584
Bacterial and virus strains		
DH5α competent cell	Tsingke	CAT# TSV-A07
Stbl3 competent cell	2nd Lab	CAT# DL1046S
Chemicals, peptides, and recombinant proteins		
Polybrene	Sigma	CAT# H9268
PES 0.45 μM filter	Millipore	CAT# SLHP033RB
TRizol reagent	Thermo	CAT# 15596018
Restriction enzymes	NEB	NA
Doxycycline hyclate	MCE	CAT# HY-N0565B
Fluorescence mounting medium	ABCAM	CAT# AB104135
PVDF	Millipore	CAT# IPVH00010
Puromycin	InvivoGen	CAT# ANT-PR-1
Aikaline phosphatase, Calf intestinal	NEB	CAT# MO290S
1% penicillin–streptomycin	Gibco	CAT# 15140122
1% non-essential amino acids	Gibco	CAT# 11140050
1% L-mlutamax	Gibco	CAT# 35050061
55 mM 2-mercaptoethanol	Gibco	CAT# 21985023
ESGRO mLIF	Millipore	CAT# ESG1107
Gelatin	Sigma	CAT# G1890
CHIR-99021	MCE	CAT# HY10182
PD0325901	MCE	CAT# HY-10254
Critical commercial assays		
Quick ligation buffer	Promega	Cat# UC6711
Improm-II reverse transcription kit	Promega	Cat# A3800
BCA protein assay kit	Beyotime	Cat# P0012
DNase I kit	Sigma	Cat# AMPD1-1KT
ThunderBird Syb Sybgreen Master Mix	Toyobo	Cat# QPK-201
iTaq™ Universal SYBR^®^ Green Supermix	Bio-Rad	Cat# 1725124
Experimental models: cell lines		
HEK-293T	Shuguo Sun’s Lab	RRID:CVCL_0063
HLF	Shuguo Sun’s Lab	RRID:CVCL_2947
Caki-1	Procell Inc	RRID:CVCL_0234
22RV1	Procell Inc	RRID:CVCL_1045
MDA-MB-231	Shuguo Sun’s Lab	RRID:CVCL_0062
MV4-11	Procell Inc	RRID:CVCL_0064
K562	Ke Tang’s Lab	RRID:CVCL_0004
E14-E14TG2a	National Collection of Authenticated Cell Cultures, China	RRID:CVCL_9108
Swiss 3T3	Shuguo Sun’s Lab	RRID:CVCL_0120
Oligonucleotides		
See Supplementary Table S1 for oligo sequences for shRNA, RT-qPCR	This study	—
Recombinant DNA		
pSPAX2	Trono Lab	—
pMD2.G	Trono Lab	—
pLenti-TRE-CasRx-U6-KDM1B-rsg1221	This study	—
pLenti-TRE-CasRx-U6-KDM1B-rsg1882	This study	—
pLenti-TRE-CasRx-U6-FKBP8-rsg433	This study	—
pLenti-TRE-CasRx-U6-FKBP8-rsg1338	This study	—
pLenti-TRE-CasRx-U6-OGT-rsg360	This study	—
pLenti-TRE-CasRx-U6-OGT-rsg2202	This study	—
pLenti-TRE-CasRx-U6-EZH2-rsg2156	This study	—
pLenti-TRE-CasRx-U6-METTL3-rsg1052 (human)	This study	—
pLenti-TRE-CasRx-U6-METTL3-rsg1604 (human)	This study	—
pLenti-TRE-CasRx-U6-Mettl3-rsg1938 (mouse)	This study	—
pLenti-TRE-CasRx-U6-DOT1L-rsg5031	This study	—
pLenti-TRE-CasRx-U6-MALAT1-rsg2836	This study	—
pLenti-TRE-CasRx-U6-MALAT1-rsg3929	This study	—
pLenti-TRE-CasRx-U6-scramble	This study	—
pLenti-TRE-CasRx-U6	This study	—
Software and algorithms		
Image Lab 5.2.1	Bio-Rad	—
Microsoft Excel 2016	Microsoft Corp	—
GraphPad Prism 8.0	Graphpad	—
FIJI 2.3.0	NIH	—
Adobe Illustrator CS6	Adobe	—
Snapgene	Snapgene	—

### Cell culture

All cells were cultured at 37°C in 5% CO_2_. HEK-293T, HLF, MDA-MB-231, and Swiss-3T3 were cultured in DMEM (Thermo, C11995500BT). K562 and 22RV1 were cultured in RPMI-1640 (Procell PM150110). MV4-11 was cultured in IMDM (Procell PM150510). Caki-1 was cultured in McCoy’s 5A medium (Procell PM150710). The medium for these cells was supplemented with 10% fetal bovine serum (PAN ST30-3302). E14 cells were cultured in DMEM supplemented with 15% fetal bovine serum (GIBCO, 141-044), 1% penicillin–streptomycin (Gibco 15140122), 1% non-essential amino acids (Gibco 11140050), 1% L-Glutamax (Gibco 35050061), 0.55 mM 2-mercaptoethanol (Gibco 21985023), 1,000 units/ml ESGRO mLIF (Millipore ESG1107), 3 μM CHIR-99021 (MCE HY10182), and 1 μM PD0325901 (MCE HY-10254) on gelatin (Sigma G1890)-coated tissue culture plates.

### Western blot

Western blot was performed exactly as previously reported (Song et al., 2021). The antibodies used are listed in the aforementioned reagent table.

### Total RNA extraction and reverse transcription

Total RNA extraction and reverse transcription were performed as previously reported (Song et al., 2021). Briefly, RNA was extracted with TRIZOL reagent following the manufacturer’s instructions and then treated with DNase I (Sigma-Aldrich AMPD1-1 KT) to remove DNA contamination. Reverse transcription was performed using the Improm-II reverse transcription kit (Promega A3800) following the manufacturer’s instructions with random primers as primers.

### Real-time quantitative PCR

Real-time quantitative PCR was performed as previously reported (Song et al., 2021). Briefly, the reaction was performed with the ThunderBird Syb Sybgreen Master Mix (Toyobo, QPK-201) or the QuantiNova SYBR Green PCR kit (QIAGEN 208054) in a 96-well plate. The primers used are listed in Supplementary Table S1.

### Cell proliferation

Adherent cells were seeded into 6-cm dishes and were transduced with lentivirus 24 h later in the presence of 8 μg/ml polybrene. MV4-11 cells were transduced with spinoculation (2000rpm, 2 h). Cells were passaged into a new 10-cm dish and selected with 1 μg/ml puromycin for 5 days. The cells were collected and stained with trypan blue and live cells were counted. Doxycycline was added to the cell culture to induce gene knockdown where indicated.

### Lentivirus production and transduction

A total of 2.6 μg psPAX2, 1.4 μg pMD2. G, and 4 μg pLenti-TRE-CasRx were co-transfected into 293T cells in a 6-cm dish. The medium was changed 24 h later. Culture suspension was collected 24 h later and filtered through a 0.45 μm filter. The suspension was then added to the cells in the presence of 8 μg/ml polybrene.

### Measuring virus infection titer

A total of 200,000 cells were seeded to a 6-well plate and transduced with different volumes of virus suspension in the presence of 8 μg/ml polybrene. The cells were passaged into a 10-cm dish 24 h later. The cells were then either let grow or selected with 1 μg/ml puromycin for 4 days and cell numbers were counted. The infection titer was calculated with the number of infected cells/volume of virus suspension.

### Immunofluorescence

Cells were fixed with 4% paraformaldehyde for 15 min at room temperature. The cells were then permeabilized with 0.5% Triton-X100 in cold PBS and blocked in 1% BSA. The cells were incubated with primary antibodies, anti-HA tag (CST #3724), washed with PBST (PBS+0.2% Tween20), and incubated with Alexa Fluor-conjugated secondary antibodies (Invitrogen #711-545-152). The cells were then stained with DAPI for 5 min at room temperature, washed with PBST, and mounted onto a slide with a mounting medium (Abcam #AB104135). Photos were taken with Zeiss-A1 Axiovert A1 fluorescence microscope equipped with a 63x oil object lens with a QImaging Retiga R6 Monochrome camera.

### Xenograft assay

Animal experiments were performed following the institute guidelines and approved by the ethics committee of Tongji Medical College and animal facility of Huazhong University of Science and Technology. Mice were kept at a temperature-controlled SPF facility at Huazhong University facility for experimental animals. The mice were acclimated to the new environment for at least 1 week. 5*10^6^ cells were injected subcutaneously into the flanks of six 5-week-old female Nu/Nu nude mice (Charles River, Beijing) in a 1:1 mixture with matrigel (BD #354248). The following day, doxycycline was added to drinking water at 1 mg/ml. Tumors were measured once a week in the beginning and twice a week later. The tumor volume was estimated by the formula 1/2*L*W*W. Before any tumor reached 1000 mm^3^, the mice were euthanized. Tumors were collected for Western blot analysis where indicated.

### Plasmid construction

The pLenti-TRE-CasRx-U6-gRNA vector was prepared by PCR, restriction digestion, and ligation. The U6 promoter and PAC were amplified from pLenti-CRISPR-V2 (addgene 52961). An SV40 early promoter was amplified from plenti4 (invitrogen). TRE was cut from pLenti6-CMV-Tight-DEST (Addgene_26433). rtTA3 was amplified and assembled from pLenti-CMV-rtTA3 Blast (Addgene 26429) and Fuw-M2-rtTA (Addgene 20342).

gRNA sequences were picked from the online source https://cas13design.nygenome.org/ (Wessels et al., 2020). Forward primers with 5′-AAAC overhang and reverse primer with 3′-AAAA overhang were annealed together and treated with polynucleotide kinase. Afterward, annealed oligo was ligated to pLenti-TRE-CasRx-U6 cut with BsmBI and treated with alkaline phosphatase. Sequences of all insertions were then verified by Sanger sequencing. Guide RNA sequences used are listed in Supplementary Table S1.

### Figure preparation

All figures were assembled in Adobe Illustrator. Bar graphs were first generated with GraphPad Prism. The map of pLenti-TRE-CasRx-U6-gRNA was first generated with Snapgene 3.2.1.

### Quantification and statistical analysis

PCR results were processed with Microsoft Excel and visualized with GraphPad Prism 8.0. Densitometry of Western blot results was performed with FIJI. For xenograft assay, two-way ANOVA was used to compare the difference.

## Results

### pLenti-TRE-CasRx-U6 expresses CasRx in a tightly inducible manner

**FIGURE 1:**
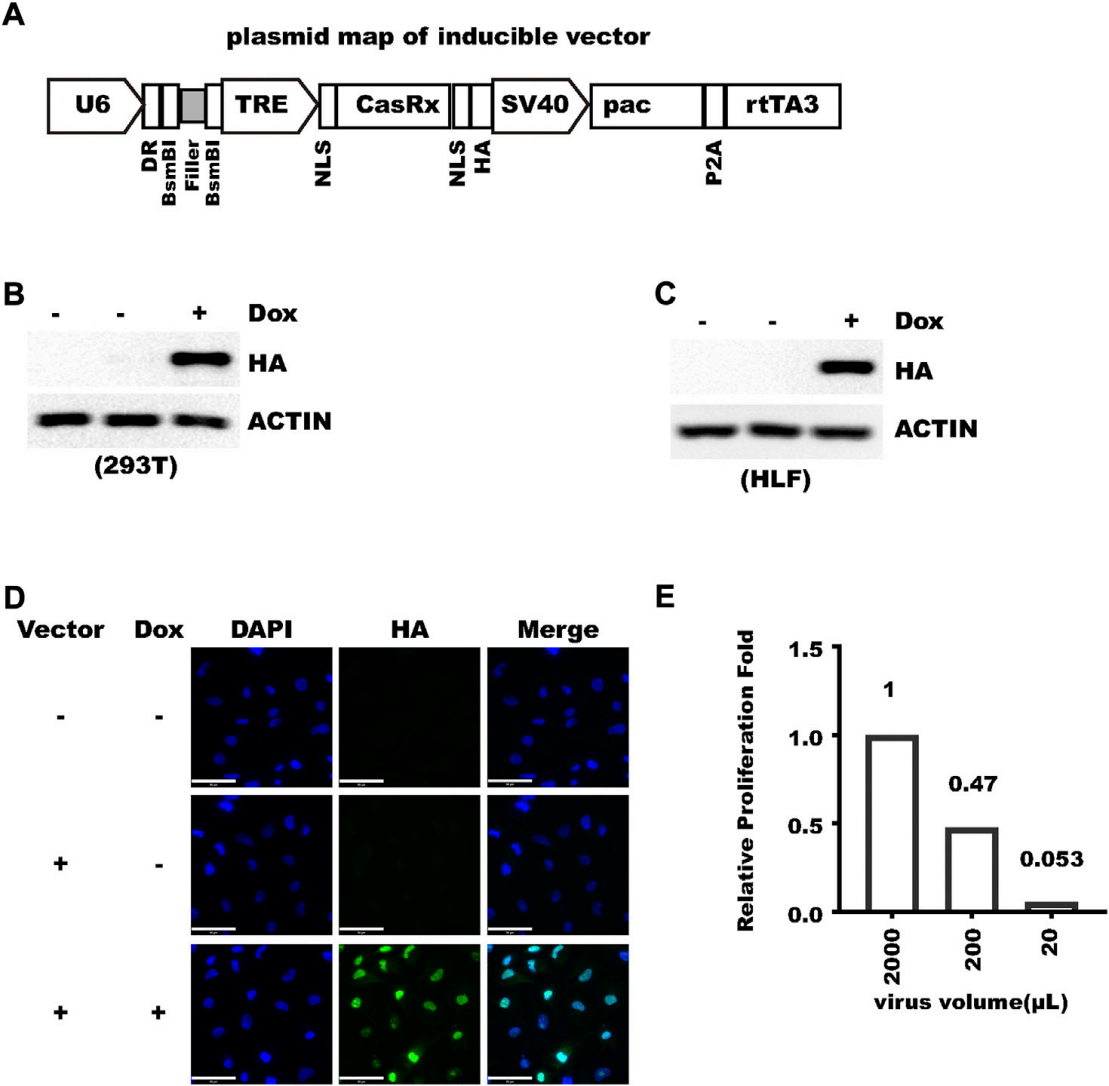
pLenti-TRE-CasRx-U6 expresses CasRx in a tightly-inducible manner. **(A)** Schematic representation of plenti-TRE-CasRx. **(B)** A total of 293 T cells were transfected with pLenti-TRE-CasRx; 24 h later, the cells were treated with 1 μg/ml Doxycycline for 24 h. Shown are WB results. First lane shows Donor cell. **(C)** HLF cells were transduced with a virus expressing pLenti-TRE-CasRx. Cells were selected with 1 μg/ml puromycin for 5 days and then treated with 1 μg/ml Doxycycline for 24 h. Shown are WB results. First lane shows Donor cell. **(D)** HLF cells were transduced with a virus expressing pLenti-TRE-CasRx; 48 h later, the cells were treated with 1 μg/ml Doxycycline for 24 h. Shown are immunofluorescence results. Nuclei were counter-stained with DAPI. Scale bar: 50 μm. **(E)** HLF cells were transduced with different volumes of virus suspension and then selected with puromycin. Cell proliferation during 4 days was counted and normalized to the cells infected with 2 ml virus which was confirmed to express HA-CasRx at about 100% with immunofluorescence.

To express crRNA and inducible RfxCas13d with one vector, we designed a compact lentivirus vector (Figure 1A and Supplementary Figure S1). In this vector, crRNA is driven by human U6 promoter and RfxCas13d is driven by a tetracycline-responsive element (TRE), which is composed of a TET-operator and minimal CMV promoter. An SV40 nuclear localization signal was fused to both the N-terminus and C-terminus to RfxCas13d to prepare the previous-named CasRx, which increased knockdown efficiency (Konermann et al., 2018). An HA-tag was fused to the C-terminus of CasRx for detection of CasRx expression. rtTA3 is driven by the SV40 promoter. SV40 also drives the expression of puromycin N-acetyltransferase (pac), which allows fast selection of transduced cells. The SV40 promoter was chosen due to its compact size and consistent activity in different cell types (Qin et al., 2010). The TRE and rtTA3 system was also shown to work in divergent systems (Qin et al., 2010). To introduce guide RNA, only the ∼23 bp sequence complementary to target RNA needs to be ligated into the vector cut with BsmBI (Figure 1A) with 5′-AAAC and 3′-AAAA overhangs. BsmBI was chosen so that sgRNA oligos inherited from the popular SpCas9-based lenti-crisprV2 (Sanjana et al., 2014) or other vectors (Wessels et al., 2020) can be directly used. After a crRNA was introduced, the size of the vector is only 10.4 kB. We name the vector pLenti-TRE-CasRx-U6-gRNA (Supplementary Figure S1). We first inserted a 23 bp oligo with no significant homology into the human/mouse genome to make pLenti-TRE-CasRx-U6-scram. With this plasmid, we then tested whether RfxCas13d expression can be efficiently induced by doxycycline. After transfected into 293 T cells, efficient induction can be detected after 24-h doxycycline treatment (Figure 1B). Robust induction was also observed with a stable cell population from human hepatocellular carcinoma HLF cells transduced with lentivirus (Figure 1C). Next, we tested whether a satisfactory virus titer can be generated with this vector and we confirmed that about 100% transduction efficiency can be readily achieved in HLF cells with virus produced from regular packaging processes even without antibiotic selection (Figure 1D). In addition, no discernible cell death was observed during 2-week puromycin selection, indicating the high efficiency of vector integration into the human genome. To measure the infection titer of the virus, we added different amounts of virus suspension to HLF cell culture and selected cells with puromycin. For HLF cells, the infection titer was calculated to be higher than 5*10^5/ml with un-concentrated 293T culture suspension (Figure 1E). Collectively, these results show that pLenti-TRE-CasRx-U6 can give rise to a satisfactory virus titer and express RfxCas13d in a tightly inducible manner.

### Knockdown can be induced in a doxycycline dosage-dependent manner

**FIGURE 2:**
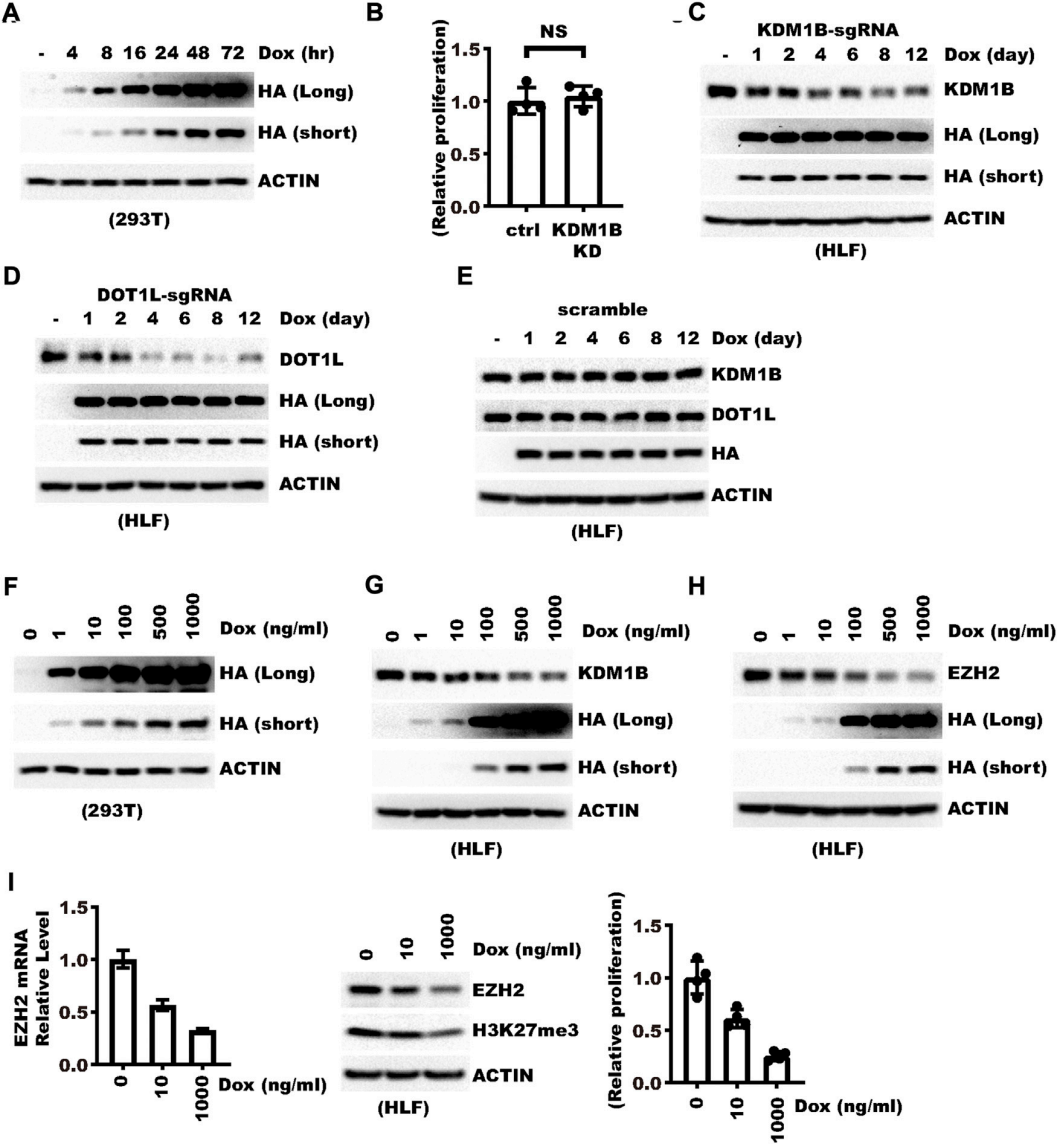
Knockdown can be induced in a Doxycycline dosage-dependent manner. **(A)** 293 T cells were transfected with pLenti-TRE-CasRx-scram. Cells were treated with 1 μg/ml Doxycycline 24 h hours later for the indicated time. Shown are WB results. **(B)** Ctrl or KDM1B-knockdown (KD) cells were seeded into a 6-well plate. Knockdown was induced with 1 μg/ml doxycycline. Shown is the relative cell proliferation during 7 days normalized to the control (Ctrl) group. “NS” denotes “not significant” as examined with a two-sided unpaired Welch’s t-test. **(C)** HLF cells were transduced with a virus expressing pLenti-TRE-CasRx-KDM1B-sgRNA. Cells were then selected with 1 μg/ml puromycin for 5 days and treated with 1 μg/ml Doxycycline for different times. Shown are the WB results. **(D)** HLF cells were transduced with a virus expressing pLenti-TRE-CasRx-DOT1L-sgRNA. Cells were then selected with 1 μg/ml puromycin for 5 days and treated with 1 μg/ml Doxycycline for different times. Shown are the WB results. **(E)** HLF cells were transduced with a virus expressing pLenti-TRE-CasRx-scramble. Cells were then selected with 1 μg/ml puromycin for 5 days. Afterward, cells were treated with 1 μg/ml Doxycycline for different times. Shown are WB results. **(F)** 293 T cells were transfected with a virus expressing pLenti-TRE-CasRx-scram. 24 h later, cells were treated for 48 h with Doxycycline at different dosages. Shown are the WB results. **(G)** HLF cells were transduced with a virus expressing pLenti-TRE-CasRx-KDM1B-sgRNA. Cells were then selected with 1 μg/ml puromycin for 5 days. Afterward, cells were treated with Doxycycline for 4 days at different dosages. Shown are WB results. **(H)** HLF cells were transduced with a virus expressing pLenti-TRE-CasRx-EZH2-sgRNA. Cells were then selected with 1 μg/ml puromycin for 5 days. Afterward, cells were treated with Doxycycline for 4 days at different dosages. Shown are WB results. **(I)** HLF cells were transduced with a virus expressing pLenti-TRE-CasRx-EZH2-sgRNA. Cells were then selected with 1 μg/ml puromycin for 5 days. Afterward, the cells were treated with Doxycycline for 4 days at different dosages. The left panel shows the relative mRNA level of EZH2. The middle panel shows the WB results. The right panel shows the relative fold of cell proliferation.

Next we applied the vector for endogenous gene knockdown and characterized the conditions for optimal effect. We first tested the time needed to induce a maximal level of RfxCas13d. In 293 T cells, a 48 h-induction with 1 μg/ml doxycycline is sufficient to induce the maximal level (Figure 2A). Next we tested the time needed to achieve optimal knockdown efficiency. We transduced HLF cells with lentivirus targeting the KDM1B gene. KDM1B was chosen for such a purpose due to our observation that knocking down this gene did not cause significant changes in HLF cell proliferation (Figure 2B). Western blot showed that 1-day induction already showed discernible knockdown while the best efficiency can be seen after four–eight days (Figure 2C). Induction beyond such time did not significantly change the efficacy (Figure 2C). A similar result was observed with another gene, DOT1L (Figure 2D). In contrast, non-targeting control sgRNA did not cause significant changes in KDM1B or DOT1L (Figure 2E). One potential advantage of the TET-On system is that Doxycycline might trigger a dosage-dependent outcome, which would be particularly advantageous over constitutive knockdown in studying the dosage effect of a gene. We first tested the effect of doxycycline dosage on CasRx expression and found that CasRx expression was induced in a doxycycline dosage-dependent manner which peaked at about 0.5 μg/ml (Figure 2F). Consistently, Western blot showed KDM1B and EZH2 knockdown efficiency also increased with the concentration of Doxycycline (Figures 2G,H). We further examined whether doxycycline generated a dosage-dependent effect downstream of EZH2. Indeed, we observed a decrease in H3K27me3 and cell proliferation concordant with Doxycycline concentration (Figure 2I), which was consistent with previous report that EZH2 could be a potential oncogene in Hepatocellular carcinoma (Chen et al., 2007). These results show that efficient knockdown can be achieved with Doxycycline induction for about 4–8 days and the efficiency of knockdown can be tuned with the concentration of Doxycycline.

### pLenti-TRE-CasRx-U6-gRNA can achieve reversible knockdown

**FIGURE 3:**
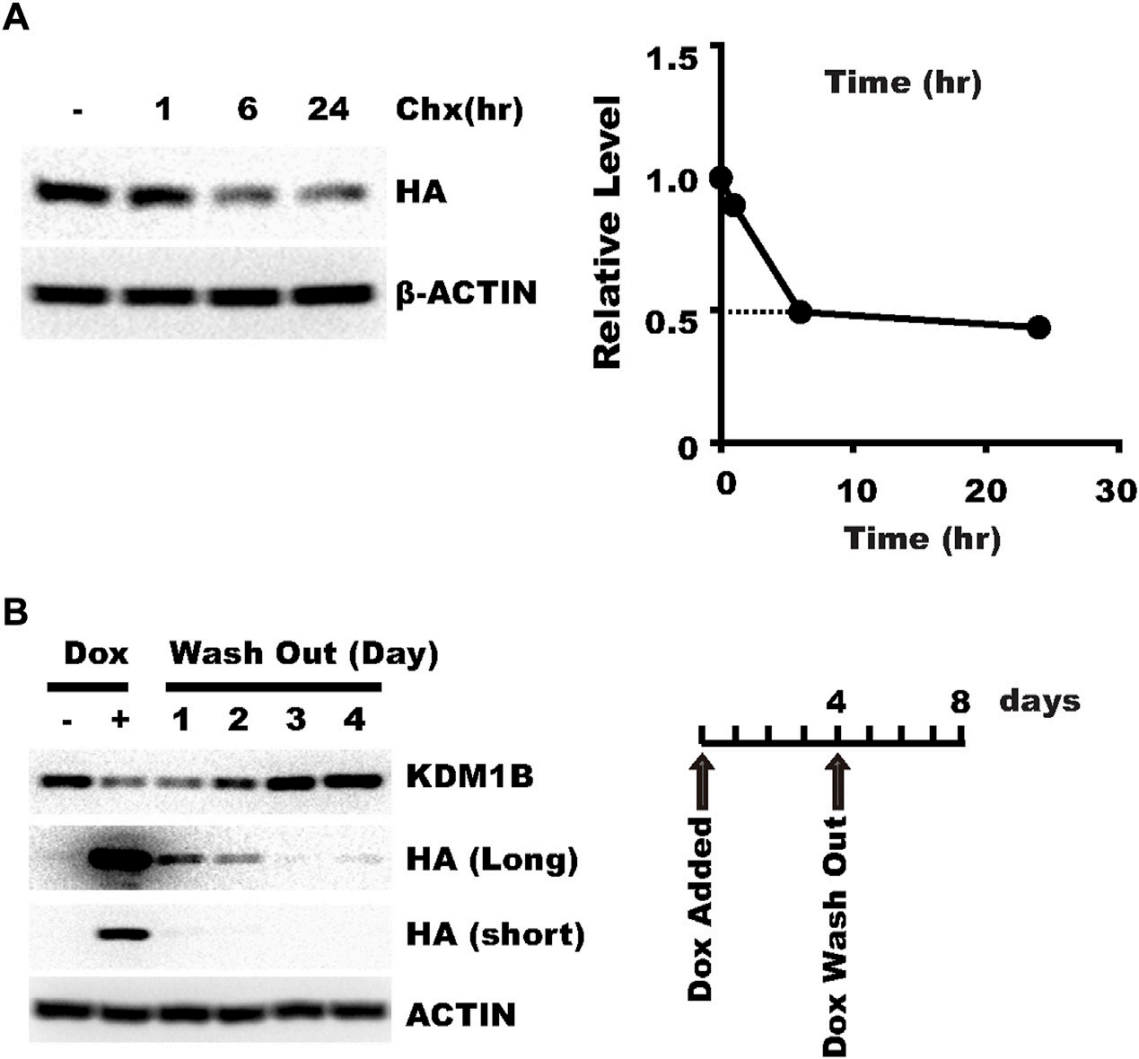
pLenti-TRE-CasRx-U6-gRNA can achieve reversible knockdown. **(A)** HLF cells transduced with a virus expressing pLenti-TRE-CasRx-scram were selected with 1 μg/ml puromycin for 5 days and then treated with 25 μg/ml cycloheximide for the indicated time. Shown are WB results. **(B)** HLF cells transduced with a virus expressing pLenti-TRE-CasRx-scram were selected with 1 μg/ml puromycin for 5 days and then treated with 1 μg/ml Doxycycline for 4 days. Cells were then cultured in a fresh medium without doxycycline for the indicated time. Shown are WB results.

Another potential advantage of the Tet-On system is that knockdown might be reversed after doxycycline withdrawal, which allows knockdown within a specific time window and determining whether the biological consequence can be reversed. One factor expected to affect the necessary time for the target gene expression to restore is the stability of the CasRx protein. So we examined the stability of CasRx with cycloheximide treatment. We found that CasRx has a fast turn-over rate in HLF cells (Figure 3A), which may presumably favor prompt target gene expression restoration after doxycycline withdrawal. Next, we washed out doxycycline in HLF cells infected with a virus targeting KDM1B. Expression of KDM1B was restored to normal 2–3 days after doxycycline withdrawal (Figure 3B). These results show that knockdown by plenti-TRE-CasRx-U6-gRNA is reversible.

### Different types of genes can be efficiently knocked-down

**FIGURE 4:**
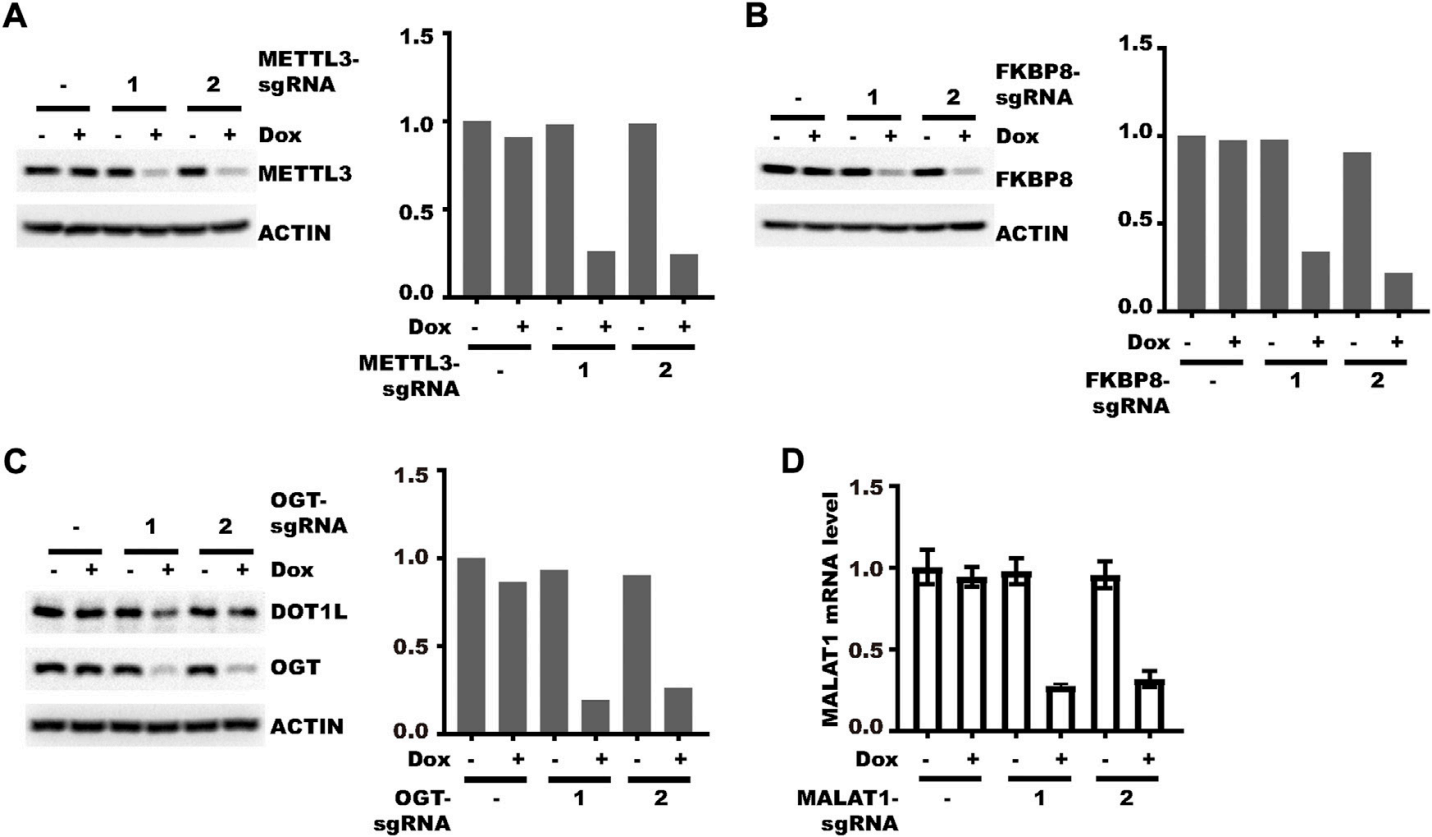
Different types of genes can be efficiently knocked-down. **(A)** HLF cells were transduced with the pLenti-TRE-CasRx-METTL3-sgRNA virus. Cells were then selected with 1 μg/ml puromycin for 5 days. Afterward, cells were treated with 1 μg/ml Doxycycline for 4 days. The left panel shows the WB results. The right panel shows the densitometric analysis of the left panel. **(B)** HLF cells were transduced with the pLenti-TRE-CasRx-FKBP8-sgRNA virus. Cells were then selected with 1 μg/ml puromycin for 5 days. Afterward, cells were treated with 1 μg/ml Doxycycline for 4 days. The left panel shows the WB results. The right panel shows the densitometric analysis of the left panel. **(C)** HLF cells were transduced with the pLenti-TRE-CasRx-OGT-sgRNA virus. Cells were then selected with 1 μg/ml puromycin for 5 days. Afterward, cells were treated with 1 μg/ml Doxycycline for 4 days. The left panel shows the WB results. The right panel shows the densitometric analysis of the left panel. **(D)** HLF cells were transduced with the pLenti-TRE-CasRx-MALAT1-sgRNA virus. Cells were then selected with 1 μg/ml puromycin for 5 days. Afterward, cells were treated with 1 μg/ml Doxycycline for 4 days. Shown are the RT-PCR results (error bars denote the standard deviation of three technical replicates).

After testing the vector as mentioned previously, we next applied it to knockdown genes of different types. We first tested its effect on protein-coding genes whose product localizes to different cellular compartments. Both nucleus-localized METTL3 (Liu et al., 2014) and cytoplasm-localized FKBP8 (Shirane and Nakayama, 2003) could be knocked-down efficiently (Figures 4A,B). OGT, a shuttling protein (Kreppel et al., 1997), can also be efficiently knocked-down, which led to a decrease in the level of its substrate DOT1L as we reported previously (Song et al., 2021) (Figure 4C). Then we examined the effect of the new vector on non-coding RNA. MALAT1, a long non-coding RNA could also be efficiently knocked-down after doxycycline treatment (Figure 4D). Collectively, pLenti-TRE-CasRx-U6-gRNA could be applied to knockdown both protein-coding genes and non-coding genes.

### Genes can be efficiently knocked-down in different cell types

**FIGURE 5:**
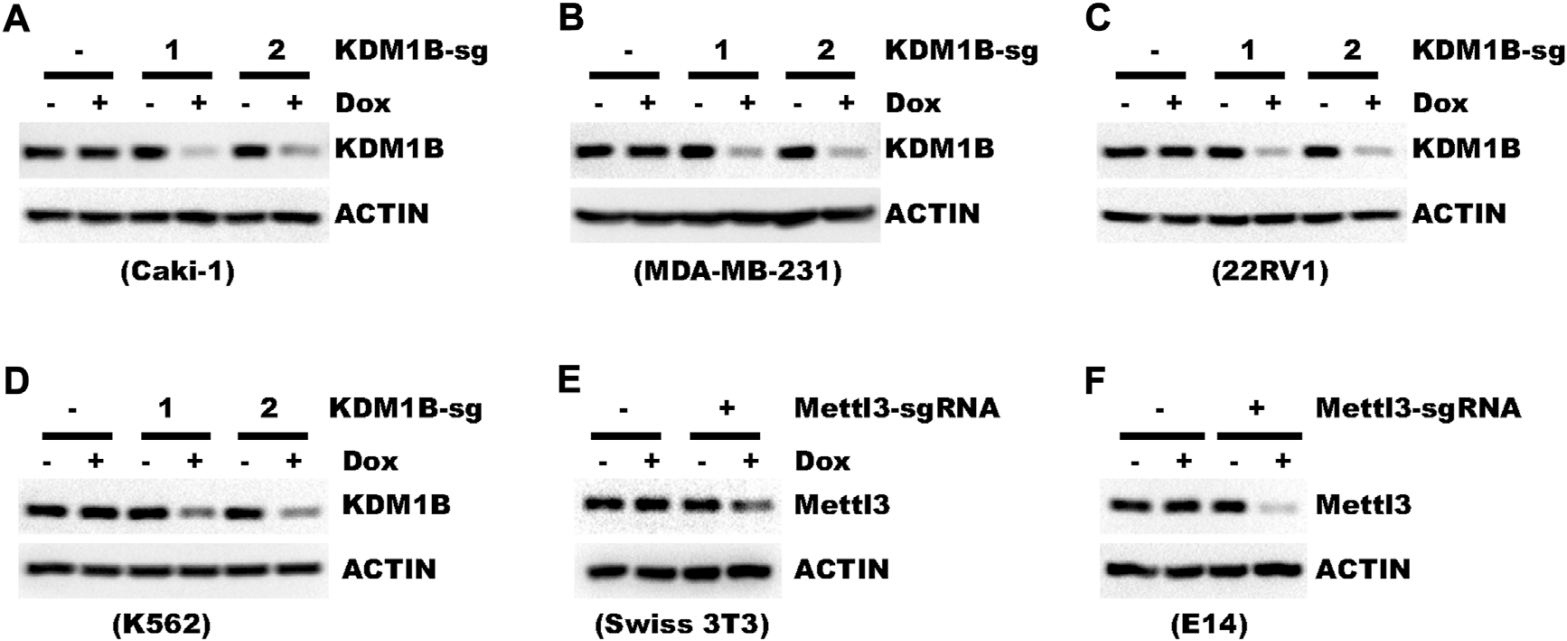
Genes can be efficiently knocked-down in different cell types. **(A–D)** Cells were transduced with a virus expressing pLenti-TRE-CasRx-KDM1B-sgRNA. Cells were then selected with 1 μg/ml puromycin for 5 days. Afterward, cells were then treated with 1 μg/ml Doxycycline for 4 days. Shown are WB results. **(E,F)** Cells were transduced with a virus expressing pLenti-TRE-CasRx-Mettl3-sgRNA. Cells were then selected with 1 μg/ml puromycin for 5 days. Afterward, cells were treated with 1 μg/ml Doxycycline for 4 days. Shown are WB results.

pLenti-TRE-CasRx-U6-gRNA requires U6, SV40, and TRE promoters all to function properly. Yet, the functionality of a promoter may vary among different cells (Qin et al., 2010). So we further tested whether pLenti-TRE-CasRx could work in different cell types. In addition to HLF liver cancer cells, crRNA targeting KDM1B led to efficient knockdown in cells of human kidney (Caki-1), breast (MBA-MB-231), prostate (22RV1), and blood origins (K562) (Figures 5A–D). We also tested the functionality of this vector in mouse cells. In both mouse fibroblast cell Swiss-3T3 and embryonic stem cell E14, Mettl3, as the target gene, was knocked-down efficiently (Figures 5E,F).

### Indispensable genes could be efficiently knocked-down in cells hard to transduce

**FIGURE 6:**
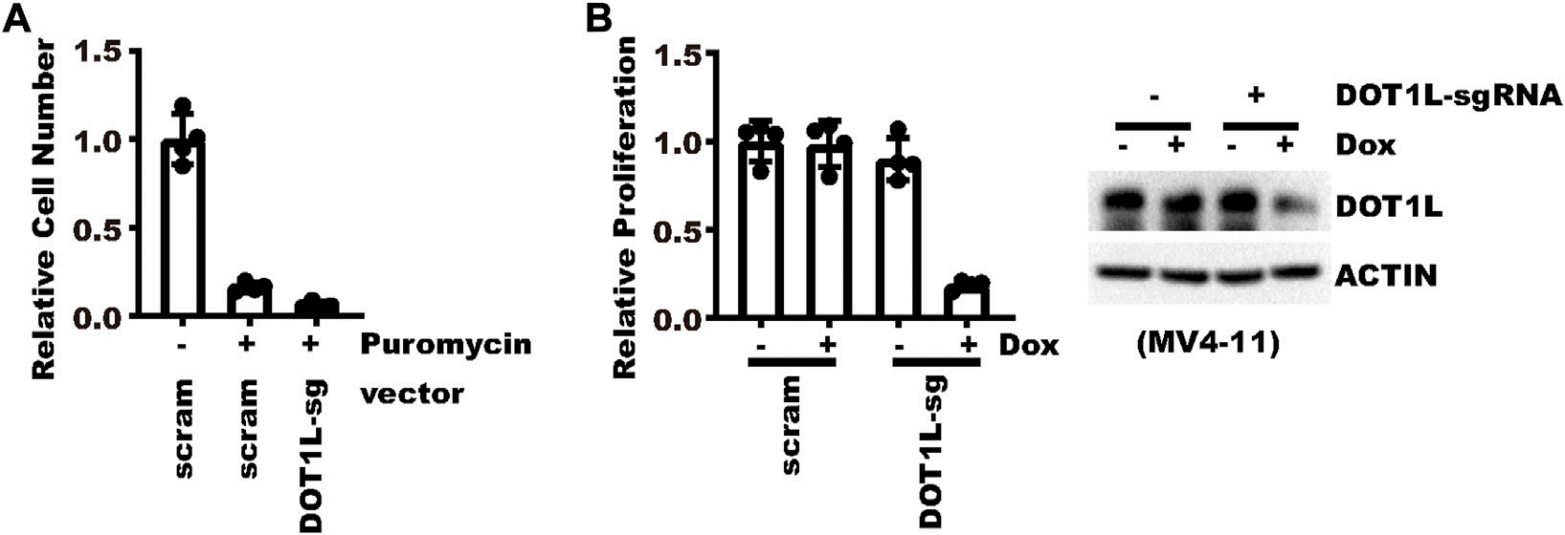
Indispensable genes could be efficiently knocked-down in cells hard to transduce. **(A)** Same number of MV4-11 cells were transduced with a virus expressing constitutive DOT1L-sgRNA or control sgRNA. Cells were then selected with 1 μg/ml puromycin for 5 days. Shown is the relative fold of proliferation after selection as compared to the group without puromycin selection. **(B)** Same number of MV4-11 cells were transduced with a virus expressing pLenti-TRE-CasRx-DOT1L-sgRNA or control vector. Cells were then selected with 1 μg/ml puromycin for 5 days. Afterward, cells were treated with 1 μg/ml Doxycycline for 7 days. The left panel shows the relative fold of proliferation after selection as normalized to the cells without Doxycycline induction. The right panel shows WB results.

In cells that are hard to transduce, an inducible vector renders further benefits over constitutive vectors, especially if knocking down a gene is detrimental to the cells. To showcase potential application in such scenarios, we tried knocking-down DOT1L in MV4-11 leukemia cells. For MV4-11, even with spinoculation, less than 20% cells can be transduced in our experiments. In addition, MV4-11 cells are very sensitive to DOT1L inhibition (Daigle et al., 2011; Daigle et al., 2013). With a constitutive vector targeting DOT1L, almost no cell was alive after 5 days of selection (Figure 6A). First, it was hard to tell how much effect came from DOT1L knockdown. Second, not enough cells were left for most mechanistic investigation. But with an inducible vector, after puromycin selection and propagation, cell proliferation was significantly inhibited by an 8-day Doxycycline treatment, which could be attributed to DOT1L inhibition (Figure 6B). Collectively, the new vector not only allows a reliable conclusion on the connection between gene knockdown and biological effects but also provides enough surviving cells for mechanistic study.

### Genes can be efficiently knocked-down *in vivo*

**FIGURE 7:**
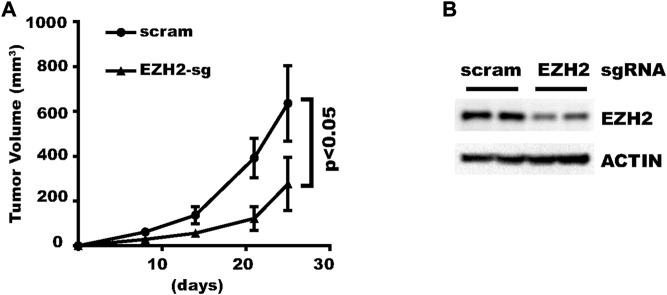
Genes can be efficiently knocked-down *in vivo*. HLF cells expressing EZH2-sgRNA or control vector was inoculated subcutaneously into nude mice. Gene knock-down was induced with Doxycycline in drinking water. **(A)** Growth curve of the xenograft tumors. **(B)** Western Blot results for four representative tumors.

After validating the efficacy of the new vector in various cell types, we then tested whether it could also work *in vivo*. We inoculated HLF liver cancer cells transduced with a virus expressing inducible scramble-sgRNA or EZH2-sgRNA. Doxycycline administered in drinking water significantly inhibited tumor growth in EZH2-sgRNA (Figure 7A). Afterward, xenograft tumors were collected and two tumors from each group were analyzed with Western blot. The result showed that EZH2 was indeed knocked down (Figure 7B). In summary, this new vector renders efficient gene knocking-down *in vivo*.

## Discussion

### pLenti-TRE-CasRx-U6-gRNA is an agile vector for CasRx-mediated gene knockdown

RfxCas13d presents a novel tool to tune down gene expression at the mRNA level with high specificity. Inducible knockdown is advantageous over constitutive knockdown in many applications. Yet current strategies rely on separate vectors to achieve inducible knockdown, which could be cumbersome to use. In this study, we present an all-in-one Tet-On vector which can achieve efficient knockdown for different gene types in different cell types and in mice. We show that the knock-down efficiency can be fine-tuned with doxycycline dosage, which may facilitate the study of the dosage effects of target genes. Importantly, we find that the CasRx protein has a short half-life in mammalian cells and knockdown mediated by this new vector can be promptly reversed by doxycycline withdrawal. Furthermore, we show this vector is particularly useful to study indispensable genes in cells hard to transduce.

As shown previously, introducing an array of gRNA separated by unprocessed direct repeat allows targeting multiple genes or one gene’s multiple regions at the same time (Konermann et al., 2018). We envisage that such applications can also be achieved with the new vector. In addition, CasRx-based vectors have been used in high-throughput screening (Wessels et al., 2020; Zhang et al., 2021). We notice an inducible system may carry extra benefits in such applications as an antibiotic selection process itself may cause the loss of some guide sequences with constitutive vectors. In addition, compared to the two-vector system, the new one-vector system might have less cell-to-cell variation in the sgRNA/CasRx ratio.

### Potential factors that might affect the efficacy

The efficiency of knockdown might be affected by many factors. We tested the effect on knockdown efficiency by Doxycycline concentration and duration. The result suggested that typically 500 ng/ml Doxycycline for 4–8 days might be enough to trigger efficient knockdown (Figure 2). We anticipate several other factors might affect the time needed to achieve optimal knockdown at the protein level. First, the abundance of the target transcript might affect the time needed to digest the mRNA. Second, higher protein stability may cause a longer time for the protein level to drop. In addition, proteins of the indispensable gene might take longer to drop for two reasons. First, dilution of the target gene product due to cell proliferation becomes limited. Second, cells with better knockdown in a population will be selected against. Despite all these potential compounding factors, we showed that robust knockdown can be achieved with this vector with various types of genes (Figure 4 and Figure 5).

### Potential adaption of the current vector

We expect that this vector can be further engineered for wider applications. First, although we have tested the efficacy of this vector in different cell types, it is possible that SV40 or human U6 promoter might not be as active in some scenarios, in which case, the promoters can be changed to a more proper one thereof. Alternatively, if a tissue-specific knockdown *in vivo* is pursed, a promoter for such a purpose could be used in place of the SV40 promoter to drive the expression of rtTA. Second, the enzymatically inactive RfxCas13d still binds the target RNA with high specificity, which has been harnessed for tracking or modifying specific RNA in the cells (Wang et al., 2019; Han et al., 2020; Xia et al., 2021; Xie et al., 2021; Xiong et al., 2021). The current pLenti-TRE-CasRx-U6 vector can also be adapted for such purposes if CasRx is replaced with its inactive mutant.

## Data Availability

The raw data supporting the conclusion of this article will be made available by the authors, without undue reservation.
