# Supplementary figures and images for: Efficient and reversible Cas13d-mediated knockdown with an all-in-one lentivirus-vector
Source: Front Bioeng Biotechnol. 2022 Sep 15;10:960192. doi: 10.3389/fbioe.2022.960192 (PMC9521038; doi:10.3389/fbioe.2022.960192)

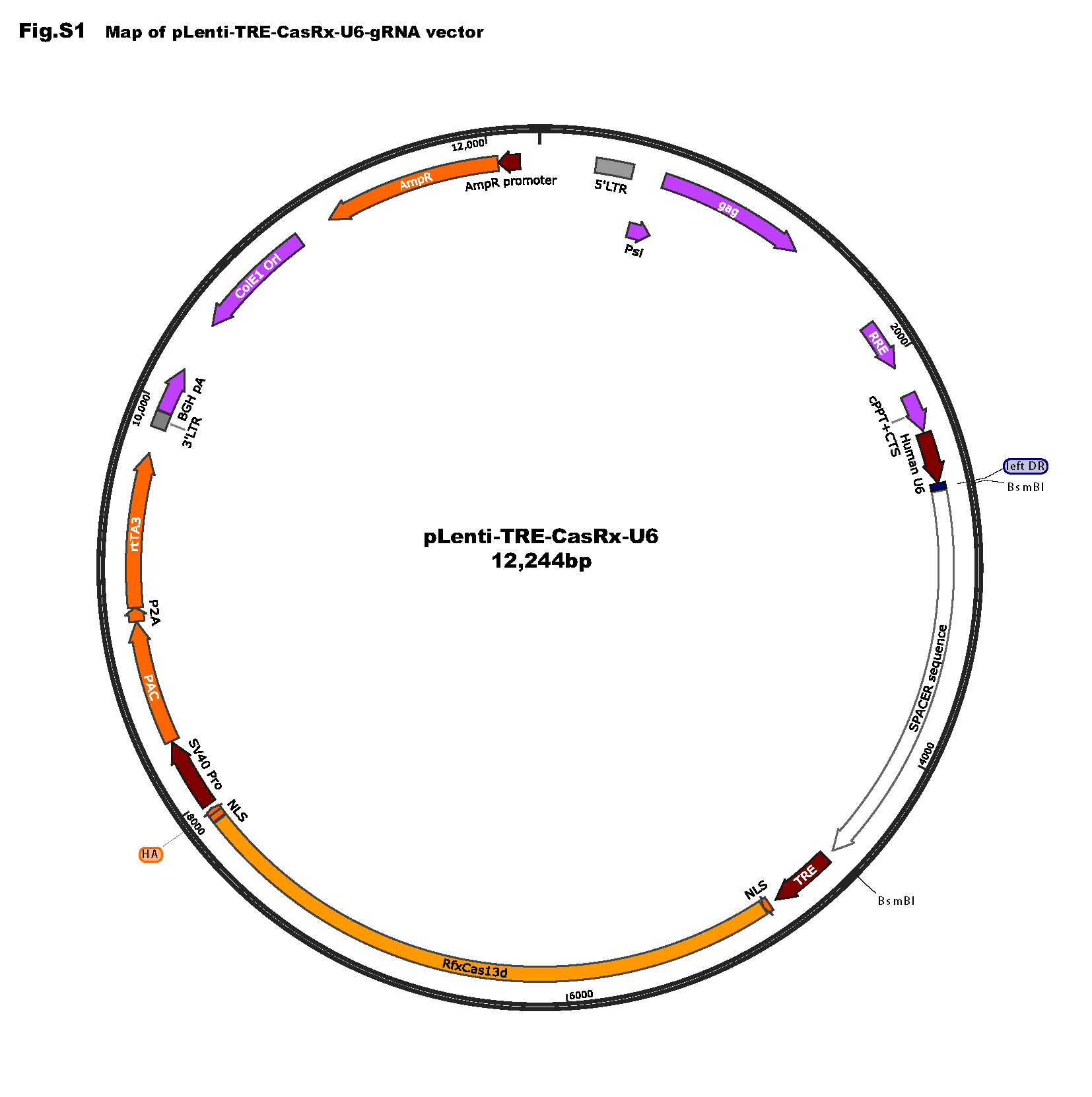

Supplement: Supplementary file 1 [file Image1.TIFF]
